# Macro Modeling of V-Shaped Electro-Thermal MEMS Actuator with Human Error Factor

**DOI:** 10.3390/mi12060622

**Published:** 2021-05-27

**Authors:** Dongpeng Zhang, Anjiang Cai, Yulong Zhao, Tengjiang Hu

**Affiliations:** 1 School of Mechanical and Electrical Engineering, Xi’an University of Architecture and Technology, Xi’an 710055, China; zdp_dp@126.com; 2 Shaanxi Key Laboratory of Nano Materials and Technology, Xi’an 710055, China; 3 State Key Laboratory for Manufacturing System Engineering, Xi’an Jiaotong University, Xi’an 710049, China; zhaoyulong@xjtu.edu.cn (Y.Z.); htj047@xjtu.edu.cn (T.H.)

**Keywords:** macro modeling, electro-thermal actuator, fuzzy mathematics calculation model, BP neural network, human error factor

## Abstract

The V-shaped electro-thermal MEMS actuator model, with the human error factor taken into account, is presented in this paper through the cascading ANSYS simulation model and the Fuzzy mathematics calculation model. The Fuzzy mathematics calculation model introduces the human error factor into the MEMS actuator model by using the BP neural network, which effectively reduces the error between ANSYS simulation results and experimental results to less than 1%. Meanwhile, the V-shaped electro-thermal MEMS actuator model, with the human error factor included, will become more accurate as the database of the V-shaped electro-thermal actuator model grows.

## 1. Introduction

Micro-Electro-Mechanical Systems (MEMS) service platforms are attractive in various applications, such as micro motors, micro robots and micro optical lens scanners [1,2,3,4,5,6,7,8,9,10]. Most MEMS service platforms are composed of MEMS actuators, flexure pivots, springs and shuttles that are designed to produce appropriate motions. V-shaped MEMS Electro-thermal actuators are typical MEMS actuators, which are widely used owing to the demands of high actuation force and accuracy [11]. However, the accurate modeling of V-shaped MEMS Electro-thermal actuators (including the electrical, thermal and mechanical mechanisms) presents a difficult problem.

The majority of the reported electro-thermal models are static [12] and computationally expensive [13]. Additionally, the human error factor does not appear in any of the articles. Due to the complexity of the manufacturing process of V-shaped MEMS actuators, the human error factor is an important factor affecting the accuracy of simulation results. The conventional manufacturing process of V-shaped MEMS actuators includes lithography and etching, both of which are subject to a large number of human errors, such as homogenizing, alignment, dry etching and wet etching. Further, the characteristics of the material itself and the operating habits of equipment also affect the accuracy of the simulation results.

This paper attempts to solve the impact of the human error factor on the accurate modeling of the V-shaped MEMS actuator. In order to develop an accurate V-shaped MEMS actuator model, BP neural network will be applied to establish a fuzzy mathematics calculation model to compare the ANSYS simulation results with the actual experimental results, with both having the same original boundary constraints. The BP neural network is a kind of multilayer feedforward network trained by an error back propagation algorithm, which is one of the most widely used neural network models at present. The BP neural network can learn and store a large number of input–output pattern mapping relationships without revealing the mathematical equations describing the mapping relationships in advance. By using the steepest descent method, the BP neural network can continuously adjust the weights and thresholds of the network through back propagation to minimize the sum of squared errors of the network.

Based on the Fuzzy Mathematics Calculation Model, the human error factor coefficient is established, which is corrected many times according to the experimental results. Considering the special fuzzy mathematics calculation modeling, the simulation results have no direct correspondence with the simulation results, except for the human error factor. The human error factor in this paper consists of personal error and systematic error. The human error factor includes all the factors that affect the difference between the experimental results and the simulation results, such as instrument error and operation error, etc. The weights and thresholds of each node of the BP network can represent various errors of the human error factor without orientation. In other words, the simulation results converted by the accurate V-shaped MEMS actuator model with the human error factor coefficient can be used as actual results in some cases when experimental conditions are not available or the cost is too high.

## 2. V-Shaped Electro-Thermal MEMS Actuator

The electro-thermal micro actuator is a typical electro-thermal-mechanical coupling system based on the Joule thermal effect and thermal expansion principle of materials. Thermal stress is the internal force of the structure. As long as the driving structure can obtain a certain amount of thermal energy, the corresponding deformation can be generated to complete the driving. Electro-thermal micro actuators are widely used in MEMS devices due to their large force.

When an electric current flows through a conductor, a certain amount of Joule heat will be generated. The heat will be transmitted outward through the three forms of heat conduction, heat convection and heat radiation. When the heat generated inside the electric thermal actuator and the heat transferred to the outside reaches a dynamic balance, the temperature distribution on the conductor will be stable, and the corresponding thermal deformation will be determined at this time.

Execution displacement is one of the key indexes to evaluate the performance of micro actuators. For electro-thermal actuators, the executive force can be defined as the external force to restore the thermal expansion deformation that has been generated to the initial state. Electro-thermal actuators have large actuators, typically in the range of a few hundred micro N to a few thousand micro N, and can produce the desired output at relatively low voltages.

### 2.1. Structure Description

**Figure 1:**
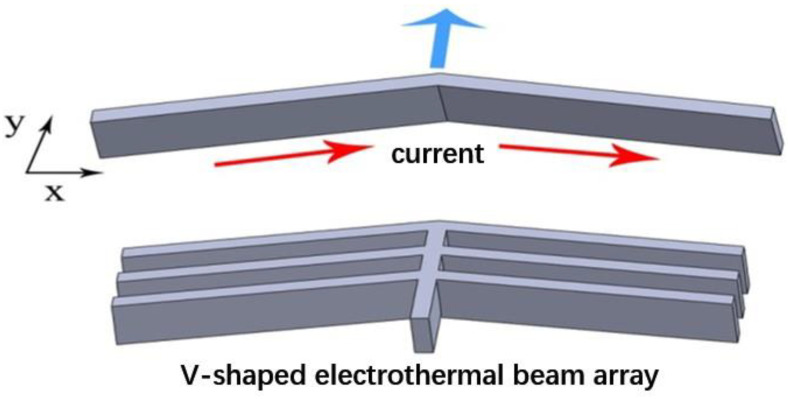
Device structure.

In this paper, the V-shaped MEMS actuator mechanism adopts a micro V-shaped electro-thermal beam structure, as shown in Figure 1. The working principle is that voltage is connected to both ends of V-shaped electro-thermal beam, and current will pass through the V-shaped electro-thermal beam, resulting in thermal expansion. Due to the symmetrical structure of V-shaped electro-thermal beam, a tiny displacement is generated toward the top angle of the beam.

### 2.2. Characterization

**Figure 2:**
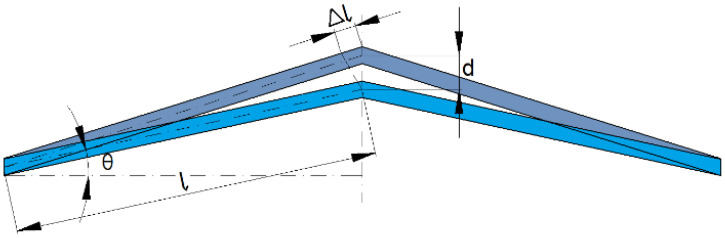
Schematic diagram of the V-shaped MEMS actuator.

The schematic diagram of the V-shaped MEMS actuator used in this paper is shown in Figure 2. 

The main parameters required in theoretical calculation and simulation (main input and output parameters) are listed in Table 1.

## 3. Modeling

The V-shaped electro-thermal beam will produce the corresponding expansion under the thermal effect, and the V-shaped electro-thermal actuator will produce a certain displacement according to corresponding executive force affected by this expansion of the V-shaped electro-thermal beam. The temperature of the beam presents an approximate quadratic distribution, and the final displacement generated by the actuator can be obtained by integrating each tiny element.

The heat generation mode of the V-shaped electro-thermal actuator is mainly the Joule heat when the current passes through the conductor according to the above discussion, and the corresponding expansion is generated. At the same time, the V-shaped electro-thermal beam generates the corresponding displacement. Thus, the main parameters can be divided into two parts: C (Original Boundary Constraints) and S (Input and Output Set), where C represents the variable set in the manufacturing process and S represents the variable set in the practical process manufacturing process. The main parameters after classification required in theoretical calculation and simulation are listed in Table 2.

The V-shaped electro-thermal MEMS actuator model, with the human error factor included, can be expressed as:

(1) $$\left[H_{C}C_{A},H_{S}S_{A}\right]=\left[C_{R},S_{R}\right]$$

$H_{C}$ is the human error factor of the Original Boundary Constraints;

$C_{A}$ is the ideal Original Boundary Constraints;

$H_{S}$ is the human error factor of the Input and Output Set;

$S_{A}$ is the simulation Input and Output Set under the ideal Original Boundary Constraints;

$C_{R}$ is the practical Original Boundary Constraints;

$S_{R}$ is the practical Input and Output Set.

The same Original Boundary Constraints are adopted in both the simulation model and the fuzzy mathematics calculation model, Therefore, the V-shaped electro-thermal MEMS actuator model, with the human error factor included, can be briefly expressed as:

(2) $$\left[C,H_{S}S_{A}\right]=\left[C,S_{R}\right]$$

C is the Original Boundary Constraints;

$H_{S}$ is the human error factor;

$S_{A}$ is the simulation Input and Output Set under the Original Boundary Constraints;

$S_{R}$ is the practical Input and Output Set.

The practical Input and Output Set can be obtained when both the human error factor and simulation Input and Output Set are known, where the human error factor can be obtained, according to the brief formulas (1) and (2), with the simulation Input and Output Set and the practical Input and Output Set (previous experimental data).

### 3.1. Simulation Model

**Figure 3:**
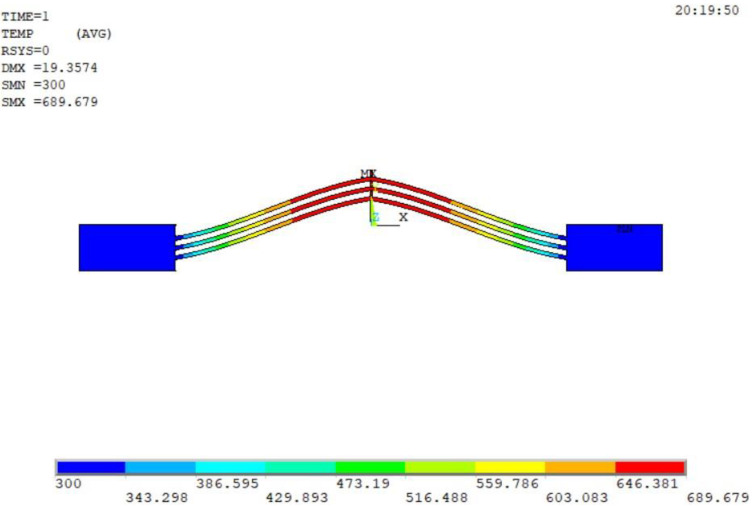
Simulation model of V-shaped electro-thermal actuator.

A detailed finite element model (FEM) of the V-shaped electro-thermal actuator is developed using ANSYS Mechanical APDL based on the structure given in Figure 3.

### 3.2. Fuzzy Mathematics Calculation Model

**Figure 4:**
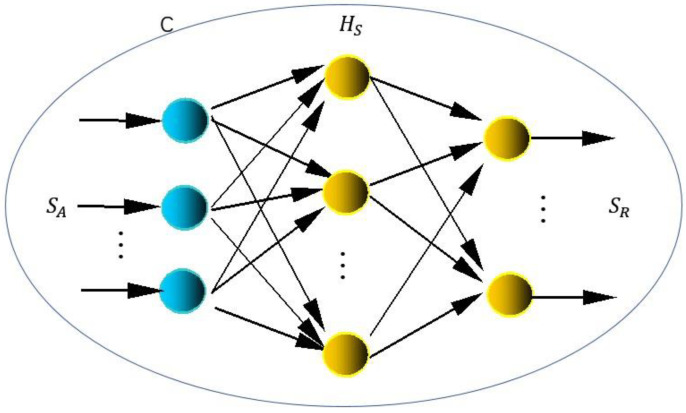
Simulation model of V-shaped electro-thermal actuator.

The BP neural network will be applied to establish the fuzzy mathematics calculation model to compare the ANSYS simulation Input and Output Set with the practical Input and Output Set, both with the same original boundary constraints. As shown in Figure 4, $S_{A}$ (simulation Input and Output Set) and $S_{R}$ (practical Input and Output Set) are input and output vectors of the network, and neurons are represented by nodes. The network is composed of the input layer, hidden layer, and nodes of the output layer. The hidden layer can be one layer or several layers (in the figure, there is a single hidden layer). The whole transformation process equation can be described as $H_{S}\;$ (human error factor).

The BP learning algorithm consists of forward propagation and back propagation. Forward propagation means that the input signal is transmitted from the input layer through the hidden layer to the output layer. If the output layer receives the desired output, the learning algorithm will end. Otherwise, the learning algorithm will switch to back propagation. Back propagation means that the error can be reduced by calculating the error (the difference between the sample output and the network output) in reverse according to the original connection path, and adjusting the weight and threshold of each layer node by the gradient descent method.

The tutor signal (the input/output sample of the network) has been given, which is the simulation Input and Output Set and the practical Input and Output Set (previous experimental data). The purpose of network training is to adjust network parameters for each input sample to minimize the output mean square error. The calculation process can be divided into the following:

1. Set the initial weight coefficient w0 as a small random non-zero value;
2. Given an input/output sample pair (tutor signal), the network output is calculated to complete the forward propagation;
3. Calculate the objective function $H_{S}$. When $H_{S}$ < ε, the training has been successfully completed and the calculation process will end; otherwise, the calculation process will continue to 4;
4. The back propagation is calculated by the output layer. The error is backpropagated by the gradient descent method, and the weight is adjusted layer by layer.

One of the most important parameters is w0, which is the accuracy of the model (the error between the simulation Input and Output Set and the practical Input and Output Set). In this paper, the default value is 1%.

## 4. Test and Discussion

Static measurements of the actuator’s tip displacement for several actuation voltages are compared with the steady-state values obtained by the simulation model, as shown in Table 3, which can be taken as the tutor signal. The Original Boundary Constraints of this dataset are listed in Table 2.

The expected value of simulation displacement at 15 V, when using the V-shaped electro-thermal MEMS actuator model with the human error factor taken into account, is 22.83 μm, and the practical displacement is 22.84 μm. The error occurs at 15 V within 1% of the practical displacement, which means that the V-shaped electro-thermal MEMS actuator model, with the human error factor accounted for, can effectively control the error between calculated value and actual results less than 1%.

## 5. Conclusions

In this paper, a V-shaped electro-thermal MEMS actuator model, with the human error factor taken into account, was proposed by means of a cascading ANSYS simulation model and a Fuzzy mathematics calculation model. The BP neural network was used to develop the Fuzzy mathematics calculation model, which can effectively correlate the simulation dataset and the experimental dataset. In addition, the human error factor is introduced into MEMS actuator model for the first time. The developed dynamic electro-thermal actuator model and experimental results are in good agreement. The V-shaped electro-thermal MEMS actuator model, with the human error factor taken into account, predicts the steady-state displacements of the V-shaped electro-thermal actuator for different voltages with a maximum error of 1%. In other words, the simulation results produced by the accurate V-shaped MEMS actuator model with the human error factor coefficient can be used as actual results in certain cases when experimental conditions are not available or the cost is too high, which means that this V-shaped MEMS actuator model, which takes into account the human error factor, has great significance in saving experimental time and cost. In addition, the model will become more accurate as the database of the V-shaped electro-thermal actuator model grows.

## Figures and Tables

**Table 1:** Basic parameters of V-shaped electro-thermal actuator.

Parameters	Item	Date	Unit
Length of beam	L	1000	μm
Width of beam	ω	40	μm
Thickness of beam	Τη	50	μm
Included angle	θ	5	°
Thickness of air film	ε	400	μm
Number of arrays	ξ	4	
Poisson ratio	λ	0.33	
Input voltage	U		V
Output displacement	D		μm

**Table 2:** Basic parameters after classification of V-shaped electro-thermal actuator.

	Parameters	Item	Date	Unit
C	Length of beam	L	1000	μm
	Width of beam	Ω	40	μm
	Thickness of beam	Tη	50	μm
	Included angle	Θ	5	°
	Thickness of air film	Ε	400	μm
	Number of arrays	Ξ	4	
	Poisson ratio	Λ	0.33	
S	Input voltage	U		V
	Output displacement	D		μm

**Table 3:** Input/output sample of the network.

Voltage (V)	Practical Displacement (μm)	Simulation Displacement (μm)	Error (%)
6	8.5	8.36	1.65
8	12.3	12.16	1.14
10	15.9	15.43	2.96
12	17.8	17.67	0.73
14	20.1	20.56	2.29

## Data Availability

Not applicable.
